# Emerging role of exosomes in vascular diseases

**DOI:** 10.3389/fcvm.2023.1090909

**Published:** 2023-03-02

**Authors:** Yi Ren, Honggang Zhang

**Affiliations:** ^1^Institute of Microcirculation, Chinese Academy of Medical Sciences & Peking Union Medical College, Beijing, China; ^2^Graduate School, Chinese Academy of Medical Sciences & Peking Union Medical College, Beijing, China

**Keywords:** exosomes, extracellular vesicles, miRNA, vascular remodeling, essential hypertension, atherosclerosis, pulmonary arterial hypertension

## Abstract

Exosomes are biological small spherical lipid bilayer vesicles secreted by most cells in the body. Their contents include nucleic acids, proteins, and lipids. Exosomes can transfer material molecules between cells and consequently have a variety of biological functions, participating in disease development while exhibiting potential value as biomarkers and therapeutics. Growing evidence suggests that exosomes are vital mediators of vascular remodeling. Endothelial cells (ECs), vascular smooth muscle cells (VSMCs), inflammatory cells, and adventitial fibroblasts (AFs) can communicate through exosomes; such communication is associated with inflammatory responses, cell migration and proliferation, and cell metabolism, leading to changes in vascular function and structure. Essential hypertension (EH), atherosclerosis (AS), and pulmonary arterial hypertension (PAH) are the most common vascular diseases and are associated with significant vascular remodeling. This paper reviews the latest research progress on the involvement of exosomes in vascular remodeling through intercellular information exchange and provides new ideas for understanding related diseases.

## Introduction

With an aging population, rapid urbanization, and risk factors such as smoking, sodium intake, elevated blood pressure, and obesity increasingly endangering cardiovascular health (1), cardiovascular disease is a major cause of morbidity and mortality worldwide (2, 3). Exploring the pathophysiology of cardiovascular disease is of great value in the search for new disease targets, biomarkers, and therapeutic approaches. Vascular remodeling is a complex adaptive process and a fundamental pathological process in cardiovascular disease. The vasculature responds to stimuli such as haemodynamic abnormalities and bioactive substances, which manifest as a series of structural and functional changes in the three components of the vascular wall, including cellular phenotypic transformation, cell migration and proliferation, and inflammatory cell infiltration (4). Communication between the adventitia, media, and intima through signals (5), such as signaling between endothelial cells (ECs) in the intima and vascular smooth muscle cells (VSMCs) in the media, is thought to be crucial for the initiation and development of pathological vascular remodeling (6); thus, vascular remodeling is a dynamic process involving the entire vessel wall.

Extracellular vesicles (EVs) are messengers of intercellular communication that carry and deliver a variety of signaling molecules, particularly proteins, mRNAs, and noncoding RNAs (7). Based on their biogenesis and size, EVs can be categorized into three main subtypes: exosomes (30–150 nm), microvesicles (100–1,000 nm), and apoptotic bodies (1,000–5,000 nm). Exosomes are intraluminal vesicles within multivesicular bodies (MVBs) that are generated by the endosomal sorting complex mechanism, microvesicles are vesicles produced by the outgrowth of cell membranes, and apoptotic bodies are fragments produced by cell collapse (8). Exosomes are capable of transmitting biological information between adjacent and distant cells (9) and are involved in the diagnosis and treatment of many diseases. Exosome-mediated intercellular communication is instrumental in maintaining homeostasis in the cardiovascular system (10, 11), and stem cell-derived exosomes have cardioprotective properties that balance efficacy and safety, indicating their potential therapeutic value (12, 13).

**Figure 1:**
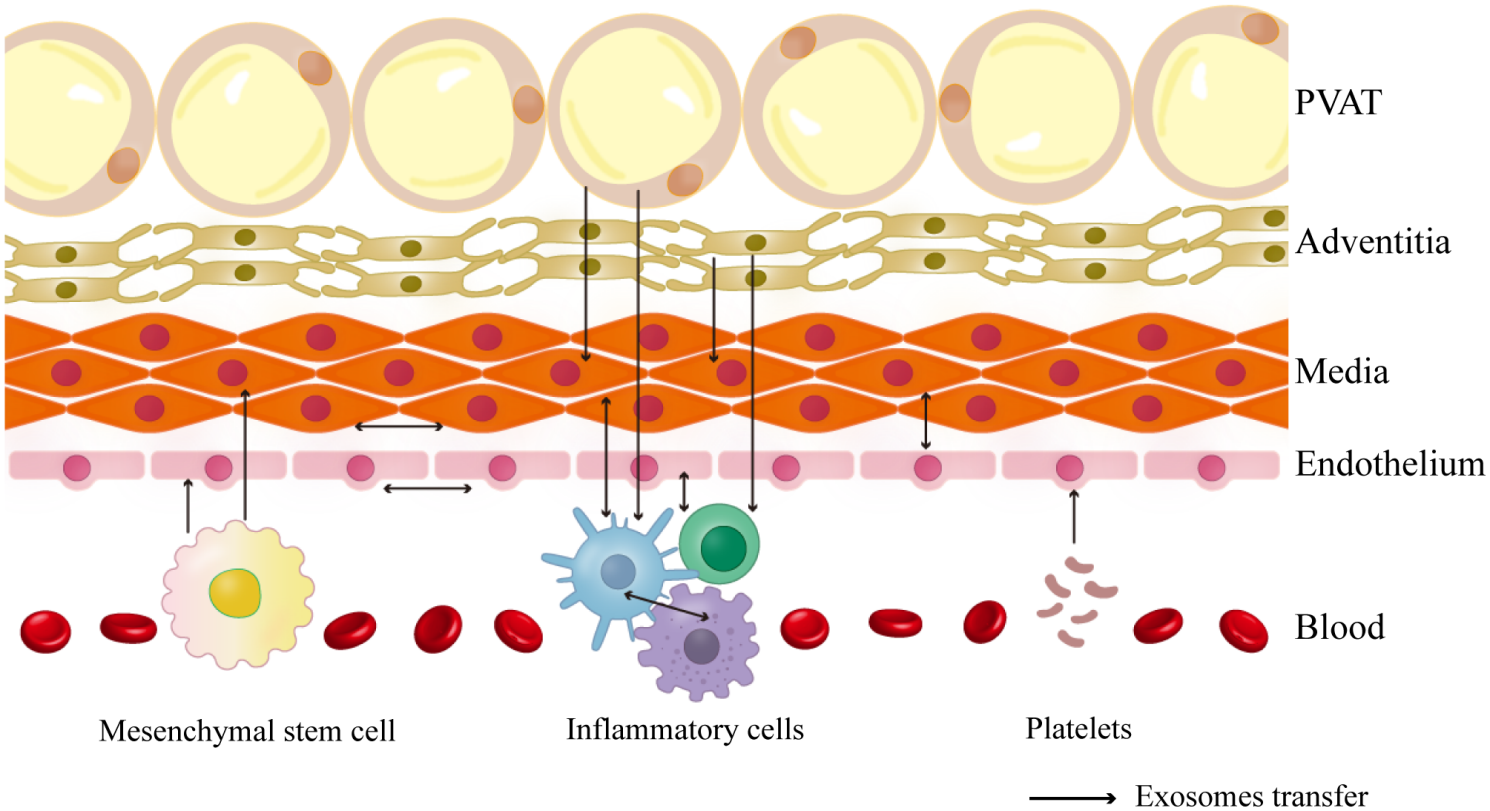
The cell-to-cell communication in the vascular environment described in this review.

Vascular remodeling involves communication between various vessel wall cells in the vascular microenvironment. This article focuses on the typical pathophysiological processes of three common vascular diseases and explores the origin and targeting of exosomes and their functions in specific cases to summarize cellular interactions during vascular remodeling (Figure 1).

## Origin and function of exosomes

Exosomes are small vesicles with lipid bilayer membranes of approximately 30–150 nm in diameter that can be released into the extracellular environment by a variety of cells, can transit through biological barriers and are extensively present in body fluids, including blood, urine, cerebrospinal fluid, and saliva (14–17). In 1985 Pan et al. (18) observed exosomes by immunoelectron microscopy and described them as small vesicles containing transferrin receptors secreted by sheep reticulocytes; most studies later concluded that exosomes are produced by the endosomal pathway (19, 20). The classic mechanism of exosome biogenesis is associated with the endosomal system; endocytosis is the initial step in which the plasma membrane invaginates into the cytoplasm to form early endosomes (EEs), which then mature into late endosomes (LEs). In EEs, NOTCH4 activation increases expression in RAB5A, whereas in the maturation process, NOTCH4 signaling is reduced, causing a reduction of RAB5A and increase in RAB7A (21, 22). Second inward invagination in LEs leads to the eventual formation of MVBs containing intraluminal vesicles (ILVs); the generation of MVBs and their intrinsic ILVs involve endosomal sorting complex required for transport (ESCRT)-dependent and ESCRT-independent machineries (23, 24). MVBs can be degraded in lysosomes or reach and fuse with the cell membrane in collaboration with microtubules, SNAREs (soluble N-ethylmaleimide-sensitive factor-attachment protein receptors), and Rab-GTPases, releasing ILVs known as exosomes into the extracellular environment (22, 25, 26). The exosomal contents are related to the location of the cell membrane and the local microenvironment at the time of exosome formation (27, 28), and exosomes also contain endogenous molecules from trans-Golgi network, endoplasmic reticulum (ER), and other organelles (29).

Many studies have shown that exosomes, microvesicles, and apoptotic bodies have distinct basic characteristics (30–34) (Table 1). However, the hypothesis that exosomes are produced only *via* the endosomal pathway is controversial. Pegtal et al. (35) suggested that exosomes are created by budding at both the plasma and endosome membranes, while Fordjour et al. (36) redirected CD63 from endosomes to the plasma membrane by eliminating its constitutive endocytosis signal in HEK293 cells and found that the budding of plasma membrane-localized CD63 remained strong, arguing against the ‘endosome-only’ hypothesis of exosome biogenesis. Due to methodological difficulties in separation and conflicting definitions, even though the term ‘exosomes’ is widely used, it has been suggested to be replaced by the term ‘small EVs (sEVs)’ according to the International Society for Extracellular Vesicles (ISEV) 2018 guidelines (37). Some EVs of nonendosomal origin have the same biophysical characteristics exhibited by exosomes (38), and it is technically difficult to effectively distinguish exosomes from other EVs, especially functional microvesicles (39). Some researchers consider ‘EV’ to be the preferred generic term, and some original studies investigated EVs, which equate to a mixture of exosomes and microvesicles. Therefore, in this review, the term ‘exosome’ is used if clarified in the referenced publication, and the term ‘EV’ is used if the differentiation is unclear.

**Table 1:** Classification of extracellular vesicles.

	Exosomes	Microvesicles	Apoptotic bodies
Origin	Multivesicular bodies (MVBs)	Plasma membrane	Plasma membrane
Regular diameter (nm)	30–150	100–1,000	1,000–5,000
Biomarkers	CD63, CD9, CD81, Alix, TSG101, HSP70	Integrins, P-selection, glycoprotein-1b, VCAMP3, ARF6	Annexin V, histones, phosphatidylserine, thrombospondin
Appearance in transmission electron microscopy	Cup-shaped	Heterogeneous	Heterogeneous
Sedimentation force (g)	100,000	10,000	2,000
Roles	Intracellular communication, material exchange between cells	Intracellular communication, transport of genetic material between cells	Regulation of pathological and physiological processes
References	(30–34)		

**Figure 2:**
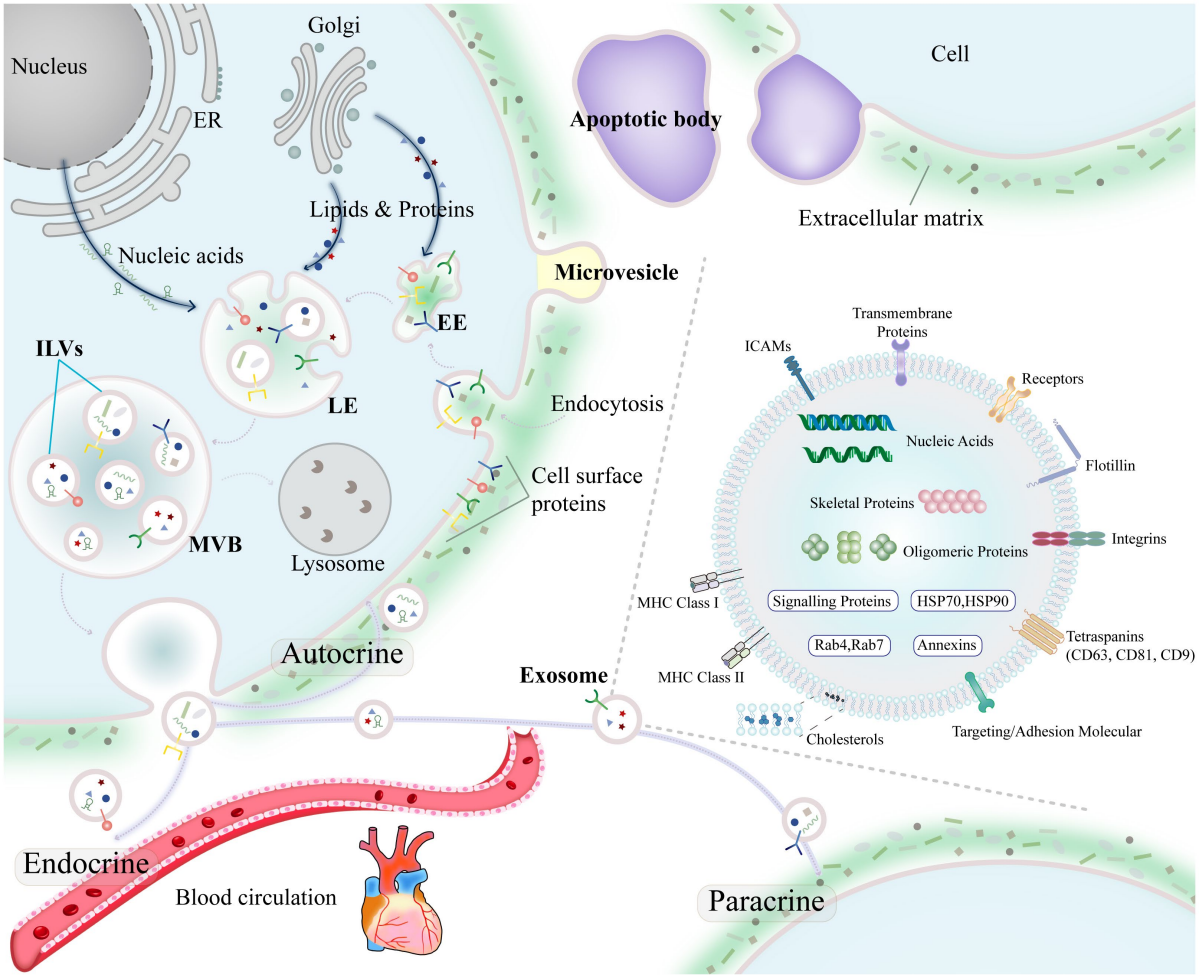
Biogenesis of exosomes and the overall composition of exosomes.

In 1996, Raposo et al. (40) isolated exosomes from human and mouse B lymphocytes by differential centrifugation and found that the exosomes could induce antigen-specific MHC class II-restricted T-cell responses. In 2007, Valadi et al. (41) showed that exosomes can deliver a specific mRNA or miRNA to recipient cells and then modify recipient cell protein production and gene expression. Exosomes are important molecules for the transmission of information between cells and are no longer thought of as simply metabolic waste excreted by cells. Through autocrine, paracrine and endocrine communication between different tissues, exosomes regulate gene expression and cellular functions (42, 43) (Figure 2). Exosomes not only regulate physiological states such as tissue regeneration, immune surveillance, and stem cell plasticity (44–46) but also participate in the pathology of diseases such as cardiovascular diseases, neurodegenerative diseases and malignant tumors (47–49) and have potential value for precise diagnosis, prognosis assessment, and disease treatment.

Almost all types of eukaryotic cells, as well as platelets, produce exosomes. For instance, mesenchymal stem cells (MSCs) have powerful paracrine activity and can secrete numerous exosomes that have been implicated in tissue repair processes and shown to have promising curative effects on cardiovascular and liver diseases (50, 51). Macrophages are widely distributed in the body and can be polarized into M1 or M2 type macrophages, and exosomes released from macrophages can modulate inflammatory signals (52). In response to an appropriate trigger, such as the activation of specific receptors or the availability of signaling factors, exosomes carry molecules from donor cells and are released into the extracellular fluid. The envelopes of the majority of exosomes can remain unbroken, are diffusible in body fluids and transfer their contents to recipient cells in diverse ways, including direct fusion with the plasma membrane of the target cell, the formation of intercellular channels with target cells using gap junction proteins, and uptake by target cells *via* the endocytic pathway (53, 54), thereby regulating recipient cell function. Furthermore, exosomes are ideal natural nanocarriers for basic research and clinical applications because of their high biocompatibility, low immunogenicity, low toxicity, circulating stability and biological barrier permeability (55). Exosomes derived from dendritic cells can induce antigen-specific T-cell responses and hold great promise in cancer immunotherapy (56, 57). Exosomes that originate from MSCs can be administered *via* intravenous or intranasal delivery; are capable of crossing the blood–brain barrier, inhibiting chronic inflammation and promoting healing and have value in the treatment of brain injuries (58). Exosomes are also candidates for regenerative cardiovascular medicine because they have lower immunogenicity and tumorigenicity and are more stabilized in the circulation than stem cells (12).

## Advances in understanding the role of exosomes in the pathophysiology of essential hypertension

Essential hypertension (EH) is one of the major risk factors for the global disease burden (59), and epidemiological investigations have shown that patients with blood pressure greater than 140/90 mmHg have significantly higher mortality rates (60). Guidelines assess patients with hypertension based on blood pressure values, as well as on multiple cardiovascular risk elements (61). The known pathophysiological alterations that occur in EH include activation of the renin-angiotensin-aldosterone system (RAAS), activation of the sympathetic nervous system (SNS), extracellular matrix imbalance, endothelial dysfunction, oxidative stress, inflammatory responses, and vascular stiffness (62). These abnormalities can work in synergy to accelerate the progression of vascular remodeling. Endothelial nitric oxide synthase (eNOS) function is uncoupled, and mitochondrial respiration produces large amounts of reactive oxygen species (ROS) (63). Then, excessive ROS production disrupts redox homeostasis, which can lead to impairment of antioxidant defense systems and a decrease in NO levels (64). In addition, the increased accumulation of ROS activates matrix metalloproteinases. The collagen and elastin content of the extracellular matrix of vascular smooth muscle is reduced, leading to vascular stiffness (65). Excessive activity of the RAAS and SNS induces structural and functional changes in VSMCs and ECs (66). Oxidative stress in the kidney and arterial wall is strongly associated with inflammatory cell infiltration, and in a salt-sensitive hypertension model, the renal tubular interstitium was infiltrated by lymphocytes and macrophages (67, 68). The intensity of inflammatory cell infiltration and the severity of blood pressure elevation are significantly correlated (69).

### Exosomes and endothelial dysfunction

Increased platelet- and endothelium-derived EVs are markers of EC activation and vascular injury (70), and several studies have focused on the alterations and importance of platelet- and endothelium-derived EVs in hypertensive states (71–73). Bao et al. (74) showed that platelet-derived EVs (pEVs), which deliver miR-92a-3p to VSMCs after intimal injury, inhibit PTEN and promote activation of the Akt pathway and the production of Col8a1 in VSMCs, leading to the deposition of Col8a1 at the site of intimal injury and increased arterial wall stiffness. Wang et al. (75) discovered that dysfunctional ECs could also secrete miR-92a-rich EVs, leading to the contractile-to-synthetic phenotype change of VSMCs or causing EC dysfunction in a paracrine manner; that circulating miR-92a levels were increased in hypertensive patients and Ang II-induced hypertensive mice compared to healthy controls; and that human plasma levels of circulating miR-92a were positively correlated with pulse wave velocity (PWV), systolic blood pressure (SBP), and diastolic blood pressure (DBP) and inversely correlated with NO levels. MiR-92a induces vascular dysfunction and is involved in the pathology of endothelial injury and atherosclerosis. However, the correlation between pathological conditions and circulating or cellular levels of miR-92a remains inconclusive, and other works suggest that miR-92a may play a protective role by maintaining vascular homeostasis. MiR-92a expression was downregulated in elderly and aged B6D2F1 mice (a model of vascular aging), which led to age-related vascular dysfunction (76). Inflammatory cell-derived exosomes have also been implicated in EC dysfunction under hypertension. Osada-Oka et al. (77) showed that Ang II-induced macrophages released less of exosomal miR-17-3p, which increased the expression of the proinflammatory factor intracellular adhesion molecule-1 (ICAM-1) in ECs.

Shang et al. (78) revealed that exosomal miR-483-3p released from ECs had a protective effect on ECs. Human umbilical vein endothelial cells (HUVECs) were incubated with Ang II *in vitro* to simulate the pathophysiological state of hypertension. MiR-483 overexpression reversed Ang II-induced changes in angiotensin-converting enzyme 1 (ACE1), endothelin-1 (ET-1), connective tissue growth factor (CTGF) and transforming growth factor-β (TGF-β) levels in ECs. Telmisartan increased miR-483-3p levels in ECs. In addition, exosomal miR-483-3p released from ECs could also be taken up by SMCs, regulating their functions and reducing hypertension-induced vascular injury by downregulating the expression levels of ACE1, TGF-β and CTGF. MiR-483-3p was also shown to protect ECs against endothelial-mesenchymal transition and inflammation in a recent study by Esmerats et al. (79). Hypertension in its early stages may increase miR-483 levels in ECs due to compensatory mechanisms, but as the disease progresses, this negative feedback effect is suppressed, and increased dysfunction occurs in ECs and SMCs, eventually leading to a decrease in miR-483-3p levels; thus, serum miR-483-3p levels are significantly reduced in patients with long-term chronic hypertension (78).

### Exosomes and the RAAS

Tong et al. (80) isolated cells from the thoracic aortas of Wistar-Kyoto (WKY) rats and spontaneously hypertensive rats (SHRs) and found that exosomes released from SHR adventitial fibroblasts (AFs) transferred ACE to VSMCs, thereby increasing Ang II levels within VSMCs and activating AT_1_R (Ang II [angiotensin II] type 1 receptor) to promote the migration of VSMCs. Subsequent studies demonstrated that miR-155-5p, which is derived from the EVs of AFs, protects the vasculature, while miR-135a-5p promotes structural changes in blood vessels. Compared to those in WKY rats, miR155-5p levels were reduced in EVs released from SHR extravascular fibroblasts. Intravenous delivery of EVs derived from WKY rats mediated miR155-5p transfer, thereby reducing ACE expression in VSMCs and the arterial blood of SHRs and attenuating vascular remodeling (81). In addition, elevated miR-135a-5p levels in the EVs of SHR AFs decreased FNDC5 expression in VSMCs (82). As FNDC5 can attenuate oxidative stress and inflammatory responses and inhibit the proliferation of VSMCs through the mitogen-activated protein kinase (MAPK) signaling pathway, miR-135a-5p delivery exacerbated VSMC proliferation and vascular remodeling (83, 84). Furthermore, Zou et al. (85) found that EVs from monocytes transferred miR-27a to ECs, decreased Mas receptor expression, reduced eNOS phosphorylation, impaired Ang-(1–7)-mediated vasodilation, and promoted hypertension.

### Exosomes and vascular calcification

Vascular calcification (VC) is an important phenotype of vascular aging that manifests not only in EH but also in atherosclerosis, chronic kidney disease and diabetes mellitus (86). Under normal physiological conditions, VSMCs exhibit a contractile phenotype and regulate microenvironmental homeostasis by actively releasing exosomes carrying endogenous inhibitors of calcification, such as fetuin A, matrix Gla protein (MGP), osteoprotegerin (OPG) and bone morphogenetic protein-7 (BMP-7), thereby inhibiting the development of VC (87). However, in pathological states, factors related to osteogenic differentiation exert their effects. For example, lipopolysaccharide (LPS)-treated macrophage-derived EVs are enriched in proinflammatory cytokines and CAD (cis-aconitate decarboxylase), PAI-1 (plasminogen activator inhibitor-1), and Saa3 (serum amyloid A-3 protein), creating an inflammatory microenvironment for surrounding cells, and these exosomes significantly increase the calcification of VSMCs by increasing the expression of osteogenic markers (osterix and osteocalcin) and decreasing the expression of a contractile marker (alpha-smooth muscle actin) (88). The mechanism by which nicotine promotes VC in clinical patients was revealed by Petsophonsakul et al. (89), who demonstrated that nicotine-induced activation of α7 and α3 nicotinic acetylcholine receptor (nAChR) increased intracellular Ca^2+^ levels and initiated the calcification of VSMCs through increased NADPH oxidase 5 (Nox5) activity, leading to oxidative stress-mediated exosomes release. A positive feedback loop between Ca^2+^, Nox5 and ROS was established that promoted VC and correlated with the action of exosomes. Specifically, Nox5-mediated oxidative stress led to the excessive production of ROS, resulting in increased intracellular Ca^2+^ and the increased release of large amounts of EVs from VSMCs; these exosomes were shown to induce calcification of the extracellular matrix (ECM) and increase the calcification of recipient VSMCs. Ca^2+^ uptake by cells *via* exosomes or Ca^2+^ channels further contributed to an increase in Nox5 activity (90). Under hyperglycaemic conditions, EV miR-32 in macrophages was upregulated and delivered to VSMCs, promoting osteogenic differentiation and inhibiting autophagy in VSMCs *via* the miR-32/Mef2d/cGMP-PKG signaling pathway (91). Lipoprotein(a) stimulation led to an increase in the annexin level in the EV cargo of primary human smooth muscle cells (SMCs) and valvular interstitial cells (VICs), and these EVs readily aggregated in the collagen matrix and induced its mineralization, leading to the formation of microcalcifications that coalesced into macrocalcifications (92). Calcifying exosomes may be involved in the formation of ectopic microcalcifications in the ECM of human arterial walls. This possibility offers a new perspective that differs from the previous suggestion that exosomes entering VSMCs alter their phenotype. Notably, warfarin increased ER stress *via* the UPR-PERK-ATF4 pathway, which further induced Grp78 to be loaded into EVs as cargo and promoted exosome release (93). Deposition of EV Grp78 in the ECM and action of Grp78 on the surface of VSMCs, i.e., the promotion of calcium phosphate crystal formation on the collagen matrix, caused VSMC calcification (93). The traditional oral anticoagulant warfarin may promote VC, whilst inhibition of ER stress represents a new therapeutic possibility.

Exosomes may also improve VC in the context of pharmacological intervention or under other conditions. For example, curcumin (CUR) and exosomes secreted by VSMCs after CUR intervention attenuated VC. *In vitro*, CUR intervention resulted in high miR-92b-3p expression in both VSMCs and exosomes and delivery of miR-92b-3p to adjacent cells, significantly reducing expression of the transcription factor KLF4 and osteogenic factor RUNX2 in VSMCs; in addition, in a rat calcification model, CUR attenuated vitamin D3-induced VC by increasing miR-92b-3p expression and decreasing KLF4 expression in the aorta (94). Melatonin induced VSMCs to produce exosomes carrying miR-204 and miR-211, which inhibited BMP-2 expression in adjacent cells in a paracrine manner and attenuated VC and senescence (95). Mansour et al. (96) found that the GFOGER peptide may be a novel and promising therapeutic target for decreasing VC, as GFOGER peptide treatment modulated the EV protein composition and inhibited the expression of osteogenic markers (Runt-related transcription factor 2, the matrix-Gla protein, osteocalcin, and tissue non-specific ALP) in VSMCs. Exosomes secreted by MSCs pretreated with advanced glycation end product-bovine serum albumin (AGE-BSA) contained a high level of miR-146a, which was transferred to VSMCs and inhibited AGE-BSA-induced calcification in a thioredoxin-interacting protein (TXNIP)-dependent manner. Thus, miR-146a-carrying exosomes may be a potential therapeutic target in VC (97). AGE stimulation resulted in the enrichment of miR-126-5P in EC-derived exosomes, which were subsequently delivered to VSMCs where they downregulated BMPR1B expression, blocking the Smad1/5/9 signaling pathway and negatively regulating calcification in VSMCs (98). The potential role for miR-126-5P in improving media calcification in the vasculature has been suggested (98).

### Exosomes and renal damage

Urinary albumin excretion (UAE) is an indicator of cardiovascular risk and renal damage in hypertensive patients, and Perez et al. (99) showed in an observational case–control study that plasma exosomal miR-126-3p and miR-26a-5p levels significantly differed between EH patients without UAE and healthy controls, and elevated plasma miR-126 levels were a marker of an increased risk of vascular injury and cardiovascular events and have potential value for early diagnosis. In addition, urinary and plasma exosomal miR-26a-5p and miR-222-3p levels were negatively correlated with UAE, and plasma exosomal miR-191-5p and miR-126-3p levels were positively correlated with the UAE rate in patients with EH plus UAE. MiR-26a-5p is an influential regulator of glomerular development and structure that protects kidney function in animal experiments (100), but its therapeutic value needs to be explored in depth. Riffo-Campos et al. (101) detected characteristic ncRNAs associated with hypertension-related UAE in urinary exosomes or plasma exosomes; among these ncRNAs were five lncRNAs (LINC02614, BAALC-AS1, FAM230B, LOC100505824, LINC01484) and miR-301a-3p, which play critical roles in regulating key pathways associated with filtration barrier integrity and tubular reabsorption and could be used to study the mechanisms of proteinuria and cardiovascular injury. Gonzalez et al. (102) studied urinary exosomal protein alterations in EH patients with renin-angiotensin system inhibition and found 21 proteins that may be associated with glycosaminoglycan degradation, coagulation and complement system abnormalities, and oxidative stress at 3 years of follow-up, and increases in urinary exosomal complement C3, complement C4a, and ceruloplasmin were predictive of *de novo* albuminuria in hypertensive patients.

## Advances in understanding the role of exosomes in the pathophysiology of atherosclerosis

Atherosclerosis (AS) is a chronic inflammatory vascular disease caused by the accumulation of lipids in the vessel wall, which leads to plaque formation and narrowing of the vessel lumen (103). AS occurs in large and medium-sized arteries with high branching and turbulence, such as the common carotid and left coronary arteries, and is the pathological basis for many fatal cardiovascular diseases (104). During AS, changes in the phenotype of the vascular system occur, including EC dysfunction, VSMC proliferation and migration, inflammatory responses, and macrophage infiltration and polarization (105). Vascular interventions, such as angioplasty and vascular bypass grafting, are commonly used to correct coronary atherosclerotic disease. However, these vascular interventions may cause damage to the vessel wall (106), and these the transfer of exosomal miR-185 to ECs was found to impair reendothelialization after vascular injury by regulating the chemokine ligand 12/chemokine receptor 4 axis (CXCL12/CXCR4) axis (107), whereas exosomal miR-21-5p activated autophagy and promoted endothelial repair by inhibiting SIPA1L2 (signal-induced proliferation-associated 1 like 2) expression (108).

### Exosomes and inflammation

Inflammation is a common mediator of many risk factors for AS, including levels of low-density lipoproteins and triglyceride-rich lipoproteins, as well as the altered behavior of cells of the artery wall. The ample preclinical implications of inflammation in AS have opened the door to many new therapeutic targets (109). Recent clinical trials have shown that targeting inflammation can reduce cardiovascular events even in individuals who have been treated with a full panel of effective standard therapies, with relevant anti-inflammatory drugs including canakinumab and colchicine (110, 111). Immune activation in AS concerns both innate and adaptive immunity, involving both immune cells and immune molecules (112). However, studies on intercellular exosome transfer have focused more on the relationship between innate immune cells and the vessel wall. In this section, we focus on the exosomes that cause arterial inflammation, which are mainly derived from inflammatory cells, ECs, and VSMCs.

#### Inflammatory cell-derived exosomes

The inflammatory response is a central part of AS, and the roles of inflammatory cells as donors or receptors of exosomes are worth exploring; several studies have confirmed that exosomes originating from inflammatory cells act on components of the vessel wall. Li et al. (113) showed that elevated levels of miR-185-3p in M1 macrophage-derived EVs increased lipid levels, inflammation, and oxidative stress in ApoE^−/−^ mice, including increased levels of cholesterol (TC), triglyceride (TG), low density lipoprotein cholesterin (LDL-C), malonaldehyde (MDA), interleukin (IL)-1β, IL-17A, and IL-23 and decreased activities of glutathione peroxidase (GSH-PX), catalase (CAT), and superoxide dismutase (SOD). MiR-185-3p affected ECs, reduces small mothers against decapentaplegic 7 (Smad7) expression, inhibited EC proliferation, and exacerbated AS development in ApoE^−/−^ mice. Other studies have shown that miR-185 induces oxidative stress-related EC death (114). Lin et al. (115) showed that the transfer of dendritic cell-derived exosomal miR-203-3p into bone marrow-derived macrophages (BMDMs) inhibited cathepsin S expression, reduced inflammatory responses, and attenuated AS progression in mice.

MiR-146a is a hallmark molecule in cardiovascular disease research, but its role in AS is controversial. Su et al. (116) showed that EVs secreted by macrophages in plaques that phagocytosed oxidized low-density lipoprotein (ox-LDL) had elevated levels of miR-146a and were delivered to macrophages to inhibit the expression of the target genes IGF2BP1 (insulin-like growth factor 2 mRNA-binding protein 1) and HuR (human antigen R or ELAV-like RNA-binding protein 1), reducing macrophage migration and promoting macrophage retention in the vessel wall. This study suggested that miR-146a in EVs accelerated the development of AS by reducing macrophage motility. However, conversely, it has been shown that miR-146a inhibits nuclear factor-κB (NF-κB) and MAPK signaling pathways by targeting TRAF6 (TNF receptor-associated factor 6), IRAK1/2 (interleukin 1 receptor-associated kinase 1 or 2), and HuR (117). Limiting the inflammatory response in various cells has been shown to reduce vascular senescence and can prevent and treat AS (118–120). It has also been shown that exosomes do not have the same effects in cells with the same receptor that are in different states. For instance, Zhong et al. (121) studied the effects of exosomal miR-146a secretion from dendritic cells. At 12 h after the first effects of exosomes from mature dendritic cells (mDC-Exos) on HUVECs were observed, the expression of adhesion molecules increased, including vascular cell adhesion molecule 1 (VCAM-1), ICAM-1 and E-selectin. Then, HUVECs were treated with mDC-Exos again for 6 h after a 24 h recovery period, and the HUVECs were shown to be resistant to the second stimulation after initial stimulation with mDC-Exos. Changes in miRNAs in HUVECs were detected, and a significant increase in miR-146a was observed. Ultimately, it was confirmed that DC-derived exosomes delivered miR-146a to HUVECs and that miR-146a protected HUVECs against secondary stimulation by inhibiting IRAK, suggesting that exosomes contribute to a negative feedback loop that regulates endothelial inflammation.

#### EC-derived exosomes

Exosomes secreted by ECs have regulatory effects on inflammatory cells. He et al. (122) showed that miR-155 was enriched in EVs released from ECs under ox-LDL induction and was subsequently transferred to THP1 cells, and these vesicles enhanced monocyte activation by shifting the monocyte/macrophage balance from the anti-inflammatory M2 type to the proinflammatory M1 type. KLF2 was found to regulate inflammation-associated miR-155 in ECs and in EVs derived from ECs. Mice that received EVs derived from KLF2-transfected ECs showed reduced AS lesions, reduced proinflammatory M1 macrophages, and increased anti-inflammatory M2 macrophages, which was at least partially caused by reduced expression of inflammation-associated miR-155. In contrast, EC-derived miR-92a could be transported to macrophages *via* EVs and reduce KLF4 levels, leading to the AS phenotype in macrophages and the formation of AS lesions (123). Wang et al. (124) demonstrated that miR-92a expression was upregulated through the ROCK/STAT3 (RhoA/Rho-associated coiledcoil forming kinase/signal transducer and activator of transcription 3) and MLCK (myosin light chain kinase)/STAT3 pathways in the AS disease state and that increased miR-92a levels reduced expression of the target gene KLF4, suggesting that the inhibition of miR-92a expression could improve vascular function. Xu et al. (125) showed that CD137-modified ECs were activated by CD137 signaling, significantly increased IL-6 levels in EC exosomes, and promoted Th17-cell responses by upregulating Akt/NF-κB signaling. High levels of IL-6 in EC-derived exosomes form a vicious cycle with the differentiation of CD4^+^ T cells toward Th17 cells, promoting the formation of AS plaques. Zhang et al. (126) infected HUVECs with an ET-1-overexpressing adenovirus (AdET-1) and observed that exosomal miR-33 levels produced by HUVECs transfected with AdET-1 were upregulated and directly affected macrophages, reducing nuclear receptor 4A (NR4A) expression and leading to proinflammatory macrophage gene activation. The harmful role of ET-1 in the development of AS is partly dependent on the regulation of macrophage polarization, which is mediated by the miR-33/NR4A axis.

#### VSMC-derived exosomes

The role of VSMCs in initiating AS and propagating lesion inflammation has been viewed as rather important, including through secretion of cytokines, acquisition of inflammatory cell-like characteristics, and foam cell formation (127). Calcium phosphate (CaP) particle deposition has been found in a number of inflammatory diseases, including AS, and small CaP particles are closely associated with VSMCs in atherosclerotic fibrous caps, affecting the proinflammatory signaling of VSMCs (128). CaP particles stimulated activation of spleen tyrosine kinase (SYK) and caspase-1 in VSMCs, leading to increased release of VSMC-derived exosomal IL-1β, highlighting the proinflammatory and pro-calcification potential of microcalcifications and VSMCs (128). Furthermore, EVs from senescent VSMCs could influence the cytokine milieu by modulating immune cell activity. Based on unbiased proteomic analysis of EVs derived from VMSCs, EVs released from senescent VSMCs induced secretion of IL-17, interferon-γ (INF-γ) and IL-10 by T cells and tumor necrosis factor-α (TNF-α) by monocytes; moreover, senescent EVs affected the differentiation of monocytes and favored mixed M1/M2 polarization with proinflammatory characteristics (129). Another study demonstrated that when VSMCs expressed more proprotein convertase subtilisin/kexin type 9 (PCSK9), the released EVs carried a proinflammatory phenotype, which reduced the migratory capacity of macrophages (130). Specifically, exposure to VSMC-EVs resulted in a significant increase in the gene expression of IL-6, VCAM-1, ET-1, ICAM-1 and E-selectin in ECs as well as a significant increase in the gene expression of CCL2 (MCP-1/chemokine (C-C motif) ligand 2), IL-1α, IL-1β, IL-6 and IL-8 in macrophages (130). Activation of ECs occurs during the initiation phase of AS, and understanding the effect of exosomes released from VSMCs on ECs may provide clues for AS treatment. VSMC-derived exosomes mediated the transfer of KLF5-induced miR-155 from VSMCs to ECs, which destroyed tight junctions and the integrity of endothelial barriers, leading to increased endothelial permeability and dysfunction (131). Blockage of the exosome-mediated transfer of miR-155 between these two cell types might serve as a therapeutic target for AS (131). The antihypertensive tripeptide Leu-Ser-Trp (LSW) could increase miRNA-145 loading in VSMC-derived EVs and attenuated ox-LDL-induced endothelial dysfunction *via* miRNA-145 delivery, which was internalized into ECs and downregulated PDCD4 (programmed cell death protein 4) expression (132).

#### Exosomes from other cells

Several studies have elucidated the possible cellular and molecular mechanisms by which dyslipidaemia promotes atherogenesis, which is associated with inflammation and EC activation. Xie et al. (133) proposed that exosomes from visceral adipose tissue in obese mice regulated the transformation and polarization of macrophage-derived foam cells, thereby promoting the progression of AS. KLF4 interacts with the coactivator p300 to regulate NF-κB activity and attenuate the expression of VCAM1 and plasminogen activator inhibitor-1 (PAI1), thereby inducing an anti-adhesive, anti-thrombotic state (134). Jiang et al. (135) showed that steatotic hepatocyte-derived EVs delivered miR-1 to ECs. MiR-1 downregulated KLF4 expression in ECs, leading to NF-κB activation and elevated expression of adhesion molecules in ECs, promoting endothelial inflammation and AS. Moreover, miR-1 has been shown to directly bind and inhibit KLF4 expression in SMCs (136). Chrysin attenuated the release of exosomal miR-92a and upregulated KLF2 expression in human coronary artery endothelial cells (HCAECs) to protect against AS (137). MiR-25-3p could also alleviate endothelial inflammation, as Yao et al. (138) showed that thrombin-induced peripheral platelet-derived exosomes (PLT-Exos) carrying miR-25-3p could be endocytosed by coronary vascular endothelial cells (CVECs) and downregulated α-smooth muscle actin by reducing Adam10 expression, inhibiting the TNF-α/NF-κB signaling pathway and collagen I a1, collagen III a1, triglyceride, total cholesterol, IL-1β, IL-6, and TNF-α levels and thereby inhibiting ox-LDL-induced inflammation and lipid deposition in CVECs. There is also a correlation between the status of donor cells and the function of the exosomes they release. Liu et al. (139, 140) suggested that perivascular adipose tissue (PVAT)-derived exosomal miR-382-5p reduced the formation of macrophage foam cells, which could be protective against AS. Exosomal miR-382-5p downregulated the expression of SR-A (scavenger receptor A) to reduce cholesterol uptake in macrophages. In addition, the overexpression of ABCA1 (ATP-binding cassette transporter A1) or ABGA1 (ATP-binding cassette transporter G1) was induced to promote cholesterol efflux. This antiatherogenic effect might be mediated by the upstream regulation of PPARγ (peroxisome proliferator-activated receptor γ). The use of PVAT-derived exosomes as a promising preventative and therapeutic strategy for AS warrants further investigation. In contrast, PVAT exosomal miR-382-5p levels were reduced in patients with coronary atherosclerotic heart disease (140). However, in obese and diabetic conditions, PVAT was shown to be dysfunctional and lose its protective effect, instead secreting proinflammatory adipokines that induce endothelial dysfunction and inflammatory cell infiltration, thus promoting AS (141, 142).

### Exosomes and cell migration

The presence of a substantial number of intimal VSMCs, such as those involved in the formation of fibrous caps, has been suggested as evidence that VSMC migration and proliferation from the media play an important role in atherogenesis (143). Burtenshaw et al. found that phosphorylation of ERK and Akt was increased in VSMCs treated with foam cell-derived EVs (FC-EVs) and stimulated VSMC migration, and the researchers speculated by proteomic analysis that proteins in EVs might have played a role in this effect (144). In addition, FC-EVs could transfer integrins β1 and α5 to the surface of VSMCs and promote cell adhesion (144). Macrophage-derived EVs containing miR-19b-3p accelerated the migration and promotion of VSMCs by targeting JAZF1, which promoted the development of AS lesions in ApoE^−/−^ mice (145). Zhu et al. (136) showed that increased levels of exosomal miR-21-3p in nicotine-treated macrophages were transmitted to VSMCs and accelerated AS progression. MiR-21-3p binding sites were present in the 3′-UTR of PTEN, and miR-21-3p promoted the migration and proliferation of VSMCs by inhibiting PTEN. Ren et al. (146) showed that M1 macrophage-derived exosomal miR-186-5p levels were increased in response to ox-LDL stimulation and acted on VSMCs; miR-186-5p downregulated SHIP2 expression, enhancing cell viability, invasiveness, and the phosphatidylinositol-3-kinase/protein kinase B/mammalian target of rapamycin (PI3K/Akt/mTOR) pathway. Inhibition of the mTOR pathway has been shown to alleviate plaque progression and play a protective role against AS (147). Other researchers identified that miR-222 originating from M1 macrophage-derived exosomes triggered functional changes in VSMCs and confirmed that miR-222 played a key role in promoting VSMC proliferation and migration by targeting cyclin-dependent kinase inhibitor 1B (CDKN1B) and cyclin-dependent kinase inhibitor 1C (CDKN1C) *in vitro* (148). Li et al. (149) demonstrated that inflammatory CD137 signaling decreased the anti-inflammatory effects of ten-eleven translocation 2 (TET2) transfer from ECs to VSMCs, thereby accelerating VSMC proliferation and migration *in vitro* and neointimal formation *in vivo*. PVAT was shown to produce EVs containing miR-221-3p, which are subsequently taken up by neighboring VSMCs, leading to dramatically enhanced VSMC proliferation and migration (150). Mechanistically, EV-mediated miRNA-221-3p transport suppressed the downstream target PGC-1α (peroxisome proliferator–activated receptor γ coactivator 1α), a known metabolic modulator of VSMCs (150).

Co-incubation of oxidized low-density lipoprotein (ox-LDL) with human mononuclear cell lines (THP-1) can model foam cells in AS pathology. *In vitro*, exosomal lncRNA LIPCAR could be transferred from ox-LDL-treated THP-1 cells to VSMCs, resulting in the upregulation of CDK2 and PCNA, which remarkably promoted the viability and migration of VSMCs (151). Another study showed that miR-106a-3p was increased in exosomes from ox-LDL-induced THP-1 cells and could promote cell proliferation and repress cell apoptosis of VSMCs (152). It was proposed that exosome-derived miR-106a-3p could directly bind CASP9 and repress the caspase signaling pathway in VSMCs (152). In contrast, studies suggesting that exosomes may inhibit VSMC migration are uncommon. Exosomal LINC01005 from ox-LDL-treated ECs promoted VSMC phenotype switching, proliferation, and migration by regulating the miR-128-3p/KLF4 axis (153). KLF4 silencing and miR-128-3p mimic alone abrogated the ox-LDL-Exo-mediated promotion of VSMC phenotype switching, proliferation, and migration (153).

### Exosomes, plaque instability and thrombosis

Hyperglycaemia is known to increase haemopoiesis in the bone marrow, thereby increasing the levels of monocytes and neutrophils, which readily enter the lesions of AS-susceptible mice and increase the burden on macrophages, accelerating AS (154). Compared to nondiabetic AS plaques, diabetic plaques are prone to becoming vulnerable AS plaques with abundant foam cells, larger necrotic core sizes, a greater extent of SMC, higher levels of macrophage apoptosis, and increased infiltration of inflammatory cells (155). Bouchareychas et al. (156) showed that exosomes from BMDMs exposed to high concentrations of glucose increased the number of hematopoietic and myeloid cells in the circulation of ApoE^−/−^ mice, leading to an increase in the number of macrophages in the lesion, as well as an increase in the area of apoptosis and AS progression. The mechanism involved the delivery of miR-486-5p *via* BMDM-derived exosomes to macrophages and a significant reduction in ABCA1 mRNA expression in macrophages. Liu et al. (157) also demonstrated that miR-486-5p reduced ABCA1 expression in THP-1 macrophages. Wang et al. (158) showed that insulin-resistant adipocytes increased the vulnerability of aortic plaques in diabetic ApoE^−/−^ mice by secreting exosomes containing sonic hedgehog (shh). The factor shh activated intracellular pathways by binding the Patched (Ptch) receptor on recipient cells (HUVECs and murine aortic endothelial cells), increasing the expression levels of TNF-α, IL-1β, IL-6, vascular endothelial growth factor A (VEGF-A), ICAM-1, matrix metalloproteinase-2 (MMP2), and MMP9 in plaques and promoting vasa vasorum (VV) angiogenesis. The VV is an important entry pathway for immune cells, promoting the development of chronic inflammatory and necrotic cores and leading to plaque instability (159).

VSMC necrosis and apoptosis play progressive roles in plaque instability and AS lesions, and the migration of VSMCs accelerates AS progression despite the possible role of these cells in stabilizing the fibrous cap (160). Wang et al. (161) showed that macrophage-derived EVs carrying miR-503-5p inhibited the proliferation, migration and angiogenesis of HCAECs while promoting the proliferation and migration of human coronary artery smooth muscle cells (HCASMCs) by downregulating Smad7, smurf1, and smurf2 and elevating TGF-β1, thereby exacerbating AS. In VSMCs, exosomal miR-223, miR-339, and miR-21 from thrombin-activated platelets inhibited the proliferation of VSMCs by reducing the expression of PDGF receptors. Because reduced PDGF receptor expression was also observed in VSMCs surrounding thrombotic areas *in vivo*, these miRNAs may be biomarkers for predicting atherosclerotic thrombosis (162). Another study showed that platelet-derived exosomes inhibited thrombogenesis in AS by reducing macrophage CD36-dependent lipid loading and inhibiting platelet aggregation (163).

## Advances in understanding the role of exosomes in the pathophysiology of pulmonary arterial hypertension

Pulmonary arterial hypertension (PAH), which is predominant in women and is defined as a resting mean pulmonary artery pressure (mPAP) greater than or equal to 25 mmHg, is a chronic and progressive cardiopulmonary disease associated with persistent perivascular inflammation, vascular remodeling and right heart failure (164–166). The activation of complement, particularly through alternative pathways, plays a key role in the early inflammatory and proliferative response in PAH (167). Irreversible remodeling of the pulmonary vascular bed is a major cause of increased mean pulmonary arterial pressure (mPAP) in PAH, and right ventricular function is a major determinant of clinical outcomes and survival in patients with PAH (168, 169). Pulmonary artery plexiform lesions are characteristic of PAH and are characterized by marked capillary hyperplasia (170). Nongenetic abnormalities associated with pulmonary hypertension include endothelial dysfunction, SMC metabolic reprogramming, perivascular inflammation, platelet and coagulation dysfunction, and extracellular matrix remodeling (168, 171–174). The pathogenesis of PAH is also associated with mutations in components of the BMP pathway, including bone morphogenetic protein receptor 2 (BMPR2), BMP9, ACVRL1, ENG, or SMAD9 (175–177), among others. Impaired BMPR2 signaling can lead to accelerated cell proliferation and mitochondrial dysfunction and promote PAH (178–180). Mutations in the BMPR2 gene are strongly associated with the development of familial PAH (FPAH) and idiopathic PAH (IPAH) (181, 182), and PAH patients with BMPR2 mutations typically have a younger onset, exhibit increased disease severity and elevated mPAP and pulmonary vascular resistance, have a poorer response to acute vasodilatory testing and are at greater risk of death (183).

### Exosomes and the BMP pathway

There is proof that BMPR2 is closely linked to miRNAs in the pathological alterations associated with PAH. A decline in BMPR-2 signaling in patients with hereditary PAH (HPAH) was shown to cause a decrease in miR-27 in ECs and an increase in translationally controlled tumor protein (TCTP) expression, resulting in EC resistance to apoptosis in patients with HPAH (184). In IPAH patients and animal models of PAH, the BMPR2 protein may be decreased even when the gene is normally expressed, indicating that the inhibition of BMPR2 transcription by miRNAs plays an important role in the pathogenesis of PAH. The depletion of miR-20a could restore BMPR2 signaling and attenuate the development of hypoxic PAH (185). IPAH patients with BMPR2 mutations have significantly more exosomes released from blood outgrowth ECs (BOECs) and contain more TCTP, which is transferred to pulmonary arterial smooth muscle cells (PASMCs), decreases caspase-3 activity and contributes to cell resistance to apoptosis (186). TCTP has potential value for diagnosing or assessing the severity of PAH. Zhang et al. (187) showed that exosomal miR-191 released from adipose-derived mesenchymal stem cells (ASCs) regulated the proliferation of pulmonary artery endothelial cells (PAECs) *in vitro*, and higher miR-191 levels were associated with faster PAEC proliferation and vice versa. In addition, miR-191 overexpression has been shown to be associated with PAH (188). MiR-191 antagonists reduced right ventricular systolic pressure (RVSP), increased BMPR2 levels in MCT-PAH rats and relieved PAH. The miR-191 inhibitor may have potential therapeutic value by preventing BMPR2 degradation to improve PAH. Zhang et al. (189) treated MCT-induced PAH mice with of MSC-derived exosomes *via* tail vein injection for 3 weeks and concluded that MSC-derived exosomes significantly reduced RVSP and the right ventricular hypertrophy index and attenuated pulmonary vascular remodeling and pulmonary fibrosis *in vivo*. *In vivo* and *in vitro* experiments confirmed that MSC-derived exosomes could regulate the Wnt5a/BMP signaling pathway in PAECs and PASMCs, including upregulating the expression of Wnt5a, Wnt11, BMPR2, BMP4, and BMP9 and downregulating the expression of β-catenin, cyclin D1, and TGF-β1.

### Exosomes and cancer-like metabolism

In PAH and cancer, mitochondrial metabolism and redox signaling are reversibly disordered, creating a pseudohypoxic redox state characterized by normoxic decreases in ROS, a shift from oxidative to glycolytic metabolism, and hypoxia-inducible factor-1α (HIF-1α) activation (190, 191), which lead to sustained ATP production, uncontrolled cell growth and cellular resistance to apoptosis (192–194). Kumar et al. (195) showed that AFs obtained from humans with PAH and calves with hypoxia-induced PAH secreted exosomes containing complement C3, which mediated BMDM activation toward a proinflammatory and metabolically altered phenotype *in vitro*, and the latter was characterized by increased aerobic glycolysis and the accumulation of succinic acid. Mitochondrial dysfunction in PAH might cause alterations in circulating exosomes, i.e., low expression of the proliferator-activated receptors gamma-coactivator 1-alpha and sirtuin 1 in exosomes, and circulating exosomes affect on other organs, such as the brain (196). Researchers observed upregulated expression of manganese SOD, downregulated expression of HIF-1α and the preservation of antioxidant enzyme expression in the brain, explaining the potential role of exosomes in PAH and antioxidant defense mechanisms in protecting cells from disease progression (196, 197). As hypoxia exposure increases, sirtuin 4 (SIRT4) gene expression also increases, and Hogan et al. (198) showed that the administration of MSC-secreted exosomes to PASMCs inhibited SIRT4 and pyruvate dehydrogenase kinase 4 (PDK4) expression, upregulated pyruvate dehydrogenase (PDH) and glutamate dehydrogenase 1 (GLUD1) expression levels, allowed glutamine and pyruvate to enter the TCA cycle and improved mitochondrial dysfunction associated with PAH. Other studies have also suggested that some exosomes may contain mitochondrial material (38, 199), but their association with PAH-related cancer-like metabolism remains to be further investigated.

### Exosomes and cell proliferation

Intercellular communication between ECs and other components of the pulmonary vascular wall is a critical signature of PAH pathogenesis (168). In an *in vitro* healing assay, Deng et al. (200) demonstrated the involvement of miR-143 in intercellular communication between PASMCs and PAECs, as miR-143 induced the migration of PAECs and reduced the death of PAECs. In a mouse hypoxia model, miR-143-3p expression was upregulated in the whole lung and right ventricle. Furthermore, histopathological and haemodynamic data collected under hypoxic conditions showed significantly reduced pulmonary vascular remodeling in miR-143^−/−^ mice compared to wild-type mice. The expression levels of miR-143-3p and miR-143-5p were also significantly upregulated in PASMCs from PAH patients compared to healthy controls. Anti-miR-143 treatment reversed hypoxia-induced RVSP elevation and right ventricular hypertrophy and reduced vascular remodeling in mice. Zhang et al. (201) showed that hypoxia increased the secretion of exosomes from PAECs to exert their proliferative effects on recipient PAECs in a paracrine or autocrine manner, which was attributed at least partially to the enrichment of lipid-peroxidizing enzyme 15-lipoxygenase2 (15-LO2) in exosomes, which then activated the STAT3 signaling pathway. Aliotta et al. (202) showed that exosomal miRNAs that were commonly increased in MCT-PAH mice and IPAH patients included the miR-17-92 cluster, miR-21 and miR-145. Among them, the miR-17-92 cluster (including miR-17, miR-18a, miR-19a, miR-20a, miR-19b-1, and miR-92-1) is a well-characterized family of miRNAs that are known to play an important role in the regulation of cell proliferation and apoptosis (202). Khandagale et al. (203) confirmed that the expression of miR-486-5p and miR-26a-5p was altered in circulating exosomes from the plasma of PAH patients and found that overexpression of miR-486-5p and downregulation of miR-26a-5p induced the proliferation of PAECs as well as the transcription and protein expression of VEGF, partly by targeting NF-κB signaling. However, some studies on myocardial infarction and cancer have shown that PTEN, PIK3R1, and MMPs may be downstream target genes of miR-26a and miR-486-5p (204–207).

Uncontrolled EC proliferation is the main contributor to the characteristic plexiform lesions of PAH, but it is undeniable that medial thickening caused by SMC proliferation is also involved in vascular remodeling in PAH (208); however, there have been few relevant exosomal studies. *In vitro*, miR-181 and miR-324 in exosomes released by KLF2-overexpressing PAECs were found to be elevated and transmitted to PASMCs, in which they alleviated hypoxia and platelet-derived growth factor (PDGF)-induced cell proliferation (209). MiR-181a-5p and miR-324-5p act together to attenuate pulmonary vascular remodeling, and their actions are mediated by Notch4, ETS1 and other key regulators of vascular homoeostasis (209). Researchers have demonstrated that therapeutic supplementation with miR-181a-5p and miR-324-5p reduced proliferative and angiogenic responses in patient-derived cells and attenuated disease progression in PAH mice (209). Zhang et al. (210) identified a role for the HIF-1-let-7b-ACE2 axis: miR-let-7b expression was elevated in rat lungs under chronic hypoxic conditions, and *in vitro* and *in vivo* experiments showed that let-7b promoted PAH by inhibiting ACE2 expression and thus inducing PASMC proliferation and migration. Nevertheless, in contrast to the above, Wang et al. (211) concluded that let-7b-5p, miR-92b-3p and miR-100-5p in human neural stem cell line (ReNcell)-derived EVs reduced vascular remodeling and RVSP in Su/Hx mice. Chen et al. (212) found that PVEC-derived EVs contained both miR-210-3P, which promoted the proliferation of PASMCs, and miR-3,057-5P, 212-5P and 34C-3P, which inhibited the proliferation of PASMCs; however, it is noteworthy that these EVs are more likely to be microvesicles than exosomes.

Pulmonary vascular ECs in PAH release specific populations of exosomes and can induce PAH alterations in healthy lungs; a positive feedback loop that propagates vascular remodeling may exist, and although it is difficult to completely reverse PAH (202), MSC-derived exosomes provide hope. Aliotta et al. (202) showed that miRNAs isolated from MSC-derived exosomes had increased levels of anti-proliferative and anti-inflammatory miRNAs, including miRs-34a, −122, −124, and −127. Other studies confirmed that MSC-derived exosomes or EVs could improve PAH by inhibiting cell proliferation and inflammatory responses. Lee et al. (213) showed that intravenous injection of MSC-derived exosomes inhibited vascular remodeling and hypoxic pulmonary hypertension. Mechanistically, MSC-derived exosomes suppressed the hypoxic activation of STAT3 and the upregulation of the miR-17 superfamily of microRNA clusters, whereas they increased lung levels of miR-204, which had pleiotropic protective effects on the lung and suppressed PAH by inhibiting the hyperproliferative pathway. MSC-derived EVs also induced macrophage polarization toward the M2 type and partially inhibited the inflammatory response of macrophages (134). Klinger et al. (214) studied a Sugen5416/hypoxia-induced PAH rat model and showed that MSC-EVs reduced macrophage recruitment, promoted a shift from an inflammatory to a reparative macrophage phenotype, promoted angiogenesis, and reduced the expression of inflammatory cytokines such as IL-6, macrophage inflammatory protein 2 (MIP-2) and TNF-α. The researchers (214) also noted that miR-196b levels were hundreds of times higher in MSC-EVs than in fibroblast-derived EVs, that HOXA5 expression was increased in PAH patients and that miR-196b reduced HOXA5 expression (215, 216). However, other studies have shown that HOXA5 can inhibit the release of inflammatory factors from macrophages (217). Therefore, it is unclear whether miR-196b plays a role in the reversal of PAH mediated by MSC-EVs. Several reports on lung injury have shown that MSC-EVs reduced macrophage recruitment in lung tissue, reduced macrophage expression of proinflammatory cytokines associated with the development of PAH, such as IL-6, MIP-2, and TNF-α, limited alveolar injury in animal models of acute lung injury and attenuated PAH in animal models of bronchopulmonary dysplasia (134, 214). In addition, Li et al. (218) showed that miR-150 prevented hypoxia-induced pulmonary vascular remodeling, fibrosis, and abnormal proliferation in PASMCs and PAECs.

## Engineered exosomes

**Figure 3:**
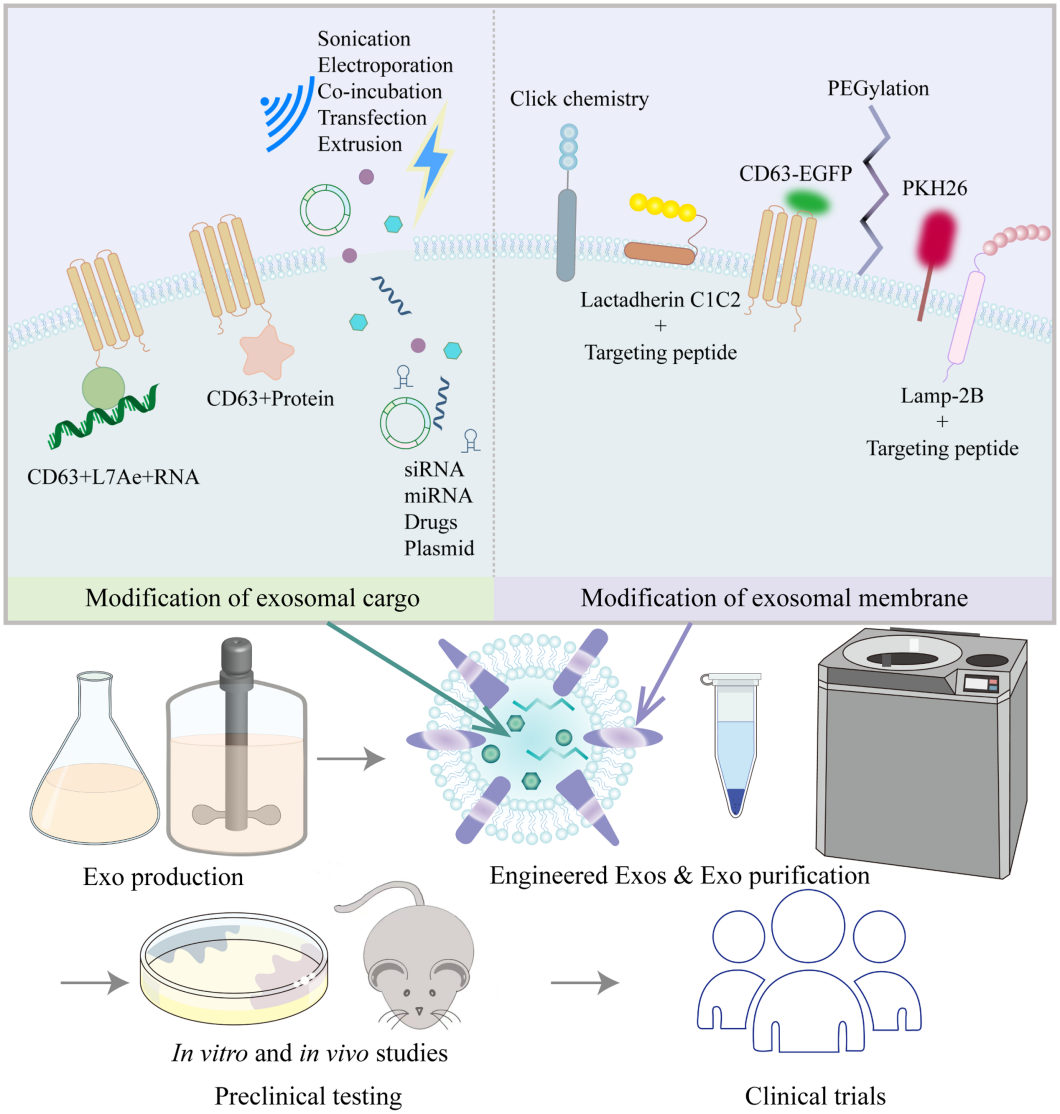
Common strategies to modify exosomes (Exos) and process of moving Exos toward clinical application.

As described above, multiple studies have shown that MSC-derived exosomes or EVs can alleviate vascular remodeling. EVs can be isolated from MSCs from various sources and can be stored efficiently, safely and easily. MSC-EVs are now known to have substantial therapeutic benefits in a range of animal disease models, and some effects have been clearly shown to be as potent as those observed in response to whole-cell MSC administration (219). Researchers have acknowledged the therapeutic value of exosomes; however, there are some challenges and confusion regarding the use of natural exosomes in disease treatment, including (220) (1) how to determine and optimize the route of administration, biodistribution, and potential toxicity of exosomes; (2) how to obtain a more comprehensive understanding of the characteristics of exosome contents to clarify the formulation and dosing regimen when using them; and (3) how to improve the targeting and accumulation of exosomes in specific organs and tissues. As a result, engineered exosomes have emerged. In addition to stem cells, cancer cells and HEK293T cells are often used as donor cells for engineered exosomes. Cancer cell-derived exosomes may have some tropism for tumors and are therefore mostly used in the field of tumor therapy (221, 222). HEK293T cells release exosomes that are almost completely nontoxic and free of immunogenicity, and the safety and efficacy of the HEK293T cell exosome drug delivery platform have been demonstrated (223, 224). Other studies have used exosomes from blood (plasma or serum), milk or plants, so the identity of the donor cells is not clear. Engineered exosomes are mainly modified by exosome content modification and exosome membrane surface modifications, which improve biological potency and targeting, enable trajectory monitoring and reduce nonspecific uptake (49) (Figure 3).

Exosome cargo modification is mainly performed to improve the biological potency of exosomes by loading them with active pharmaceutical ingredients before or after exosome isolation. Prior to exosome isolation, active packaging is mostly used to package target delivery molecules into exosomes by the transfection of target genes into cells *via* plasmids or by coexpression with exosomal membrane proteins (LAMP-2B, CD9, CD63, CD81, etc.). Chen et al. (225) transfected Lv-miR-125b into BMSCs and then collected and purified the exosomes. BMSC-Exos-miR-125b (50 μg) were injected adjacent to the coronary ligation zone in a rat model of myocardial ischaemia–reperfusion injury, which significantly increased cell viability, decreased the apoptotic ratio, downregulated Bax and caspase-3 levels, upregulated Bcl-2 expression, and decreased IL-1β, IL-6, TNF-α, and SIRT7 levels. Han et al. (226) transfected a miR-675 mimic into human umbilical cord MSCs, then isolated the exosomes and applied a silk fibroin hydrogel to encapsulate the exosomes. Laser Doppler perfusion imaging showed that delivery of miR-675-overexpressing engineered exosomes *via* a silk fibroin hydrogel improved blood perfusion in the ischaemic hind limb of senescent mice. By means of active packaging, it was possible to successfully load not only small molecules such as miRNAs, agomirs and antagomirs to the exosome cargo but also large molecules such as mRNAs and proteins. Kojima et al. (227) constructed the EXOtic device using HEK293T cells as exosome-producing cells. The researchers cotransfected the potential RNA packaging device (CD63 + archaeal ribosomal protein L7Ae + catalase mRNA) and a cytosolic delivery helper (connexin 43 S368A mutant) together with the exosome production booster and a reporter coding for encoding nluc mRNA bearing C/Dbox(es) in its 3′-UTR, as well as the targeting module RVG-Lamp2b, which was reported to target exosomes to the brain by binding the nicotinic acetylcholine receptor (CHRNA7). By analyzing the expression levels of several markers (GFAP, Iba1, TNFα, CD11b), the engineered exosomes containing peroxidase were shown to attenuate 6-hydroxydopamine (6-OHDA)-induced neuroinflammation in mice, providing a new strategy for the treatment of Parkinson’s disease.

After the isolation of exosomes, passive packaging approaches have been mostly used to package target delivery molecules into exosomes by electroporation, sonication, co-incubation, extrusion, and freeze–thaw cycles, etc. Through passive packaging approaches, some studies have successfully added small molecules such as siRNAs, miRNAs or drugs to exosome cargoes. Zhao et al. (228) used incubation and extrusion to load siS100A4 into exosomes, which were designed to have high affinity for the lung and exert prominent gene silencing effects, significantly inhibiting lung metastasis in a mouse model of postoperative breast cancer tumor growth. Sun et al. (229) isolated exosomes from plasma and introduced miRNA-21 analogs into the exosomes by electroporation. The modified exosomes were then injected *via* the tail vein into a doxorubicin-induced cardiotoxicity mouse model, and ultrasound-targeted microbubble disruption was used to facilitate exosome delivery to the cardiac region. Echocardiography confirmed that exosomal miRNA-21 delivery improved doxorubicin-induced cardiac dysfunction. Kim et al. (230) carried out *in vivo* trials of non-small cell lung cancer treatment by loading paclitaxel into macrophage-derived exosomes by ultrasound treatment. Kalani et al. (231) loaded CUR into embryonic stem cell exosomes (MESC-Exos) by repeated rapid freeze–thawing. After intranasal administration, the modified exosomes alleviated inflammation, reduced astrocytic GFAP expression, and reduced neuronal NeuN expression in the cerebral cortex and hippocampus of mice with middle cerebral artery ischaemia–reperfusion injury. Qu et al. (232) applied saturated solution incubation to load dopamine into blood exosomes, and the modified exosomes could transport drugs across the blood–brain barrier *via* transferrin receptor-mediated endocytosis, resulting in a more than 15-fold increase in brain distribution of dopamine, which is important for the targeted treatment of Parkinson’s disease and other central nervous system disorders.

Exosomal membrane surfaces have been modified in a variety of ways, mainly for the purpose of improving targeting and tracking. Xu et al. (233) fused MSC-binding peptide E7 to the exosomal membrane protein Lamp-2b, giving exosomes the ability to target synovial fluid-derived MSCs. KGN delivered by these modified exosomes was able to efficiently enter SF-MSCs and strongly induce chondrogenic differentiation while protecting chondrocytes from degeneration. Wang et al. (234) genetically modified the parent cells of EVs by fusing the ischaemic myocardium-targeting peptide CSTSMLKAC with the EV membrane protein Lamp-2b to enhance the specificity and efficiency of EVs in targeting the ischaemic myocardium. Tian et al. (235) isolated EVs from a human neural progenitor cell line and constructed a recombinant fusion protein with neurological targeting properties on EV membranes: an arginine-glycine-aspartate (RGD)-4C peptide (ACDCRGDCFC) fused to the phosphatidylserine (PS)-binding domain of lactamycin-C1C2. For *in vivo* validation in middle cerebral artery occlusion (MCAO) mice, RGD-C1C2-binding EVs (RGD-EVs) were injected *via* the tail vein, and the modified EVs were shown to effectively target ischaemic brain lesions and strongly inhibit the inflammatory response. Polyethylene glycol modification increased the circulation time of exosomes and reduced their uptake by nonspecific cells (236). Aminoethylbenzamide-polyethylene glycol-modified exosomes (AA-PEG-exoPTX) carrying paclitaxel (PTX) had high drug loading and strong accumulation capacity in cancer cells after systemic administration, showing high anticancer efficacy in a mouse model of metastatic lung cancer (230). Different techniques for exosome labeling include fluorescent labeling, bioluminescent labeling and radioisotope labeling. Lipophilic dyes include Exoria, PKH67, PKH26, carbocyanine dyes (DiI, DiO), FM4-64, and others (237–239). Each dye has its own characteristics; for example, PKH-26 has a long half-life (238), and Exoria is suitable for labeling MSC-EVs (237). Fluorescent dyes are embedded in the bilayers of exosomes with noncovalent bonds; however, these dyes can form aggregates or micelles in aqueous solutions in similar proportions to exosomes, which may give misleading information in absorption experiments (240). Targeted metabolic labeling techniques and click chemistry approaches can likewise aid exosome tracing. Angiogenesis is characterized by the over-expresses ανβ3 integrin, which plays a key role in endothelial cell survival, migration and angiogenic growth, and ανβ3 integrin can be specifically bound by a peptide containing the RGD sequence. RGD-labeled exosomes containing unnatural monosaccharide derivatives (Ac_4_ManNAz) were delivered into HUVECs in a receptor-mediated manner, which revealed that unnatural sialic acids with azide groups were artificially generated on the cell surface. The fluorescent dye DBCO-Cy3 (dibenzylcyclooctyne-cyanine3) was strongly and specifically bound to the unnatural sialic acids outside of the cell surface by copper-free click chemistry for glycan imaging (241, 242). Click chemistry has also been used to improve exosome targeting, and thiol-maleimide cross-linking is one of the most classical and established click chemistry methods. To allow prostate cancer cells to efficiently recognize and internalize exosomes, Han et al. (243) used the thiol-maleimide cross-linking reaction to generate E3 aptamer-PEG-cholesterol adducts to modify the exosome surface. In addition, labeling could be achieved by generating CD63EGFP fusion proteins, such as by cotransfecting stable cell lines using the PCL6-CD63EGFP plasmid, which resulted in CD63-EGFP fusion protein-tagged exosomes, were then analyzed by serial two-photon tomography (STP) and anti-EGFP antibody staining (244, 245). Among the bioluminescent markers, exosomal surface-expressed reporter proteins such as Gaussia luciferase, firefly luciferase or kidneyworm luciferase produced bioluminescence when their substrates, such as cecropin (CTZ), were added (246). Radioisotope labels such as ^131^I, ^99^mTc, and ^111^In-oxine are often used to label exosomes and allow the imaging of deeper tissues due to their tissue penetration potential, and these labels are commonly used for assessing exosome biodistribution in isolated organs (247, 248).

Preclinical studies and clinical trials involving exosomes or EVs continue to emerge. According to a systematic review in 2021, more than half of preclinical studies on EV-delivered drugs in the last decade have focused on cancer therapy, and 12.7% involved cardiovascular disease (247). We searched ‘exosome’ or ‘extracellular vesicle’ at https://www.clinicaltrials.gov, and 191 related trials are active and recruiting. However, it is important to note that there is a lack of quality control standards, as well as evaluation criteria for the use of engineered exosomes for drug delivery or disease treatment (249).

## Conclusion

Exosomes are widely involved in the development and progression of EH, AS and PAH and play an influential role in vascular remodeling. Exosomal miRNAs hold particular promise for the development of new tools for the diagnosis and treatment of vascular diseases.

**Figure 4:**
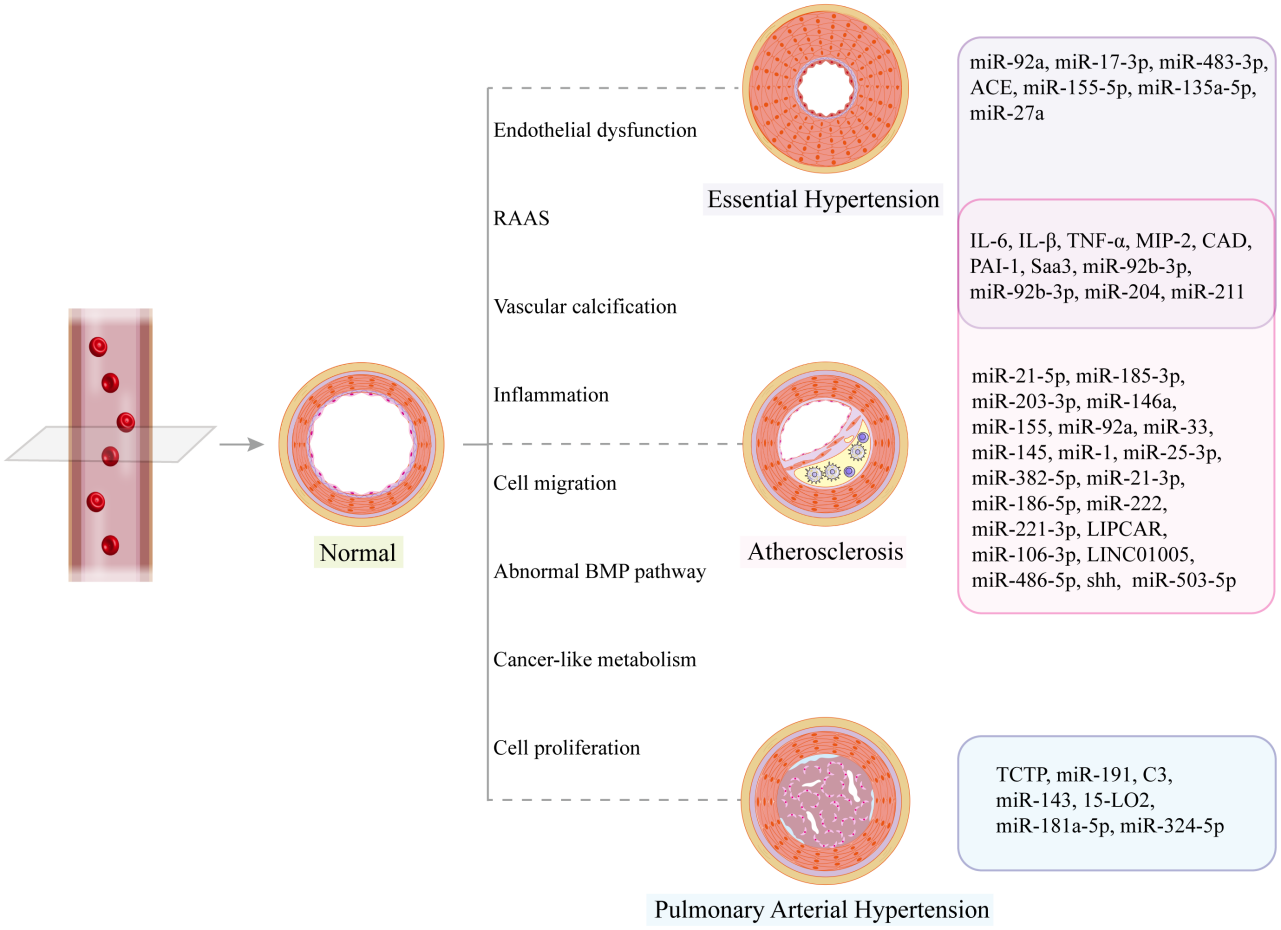
Vascular structural changes may occur in disease states, and related exosome cargos can promote or alleviate vascular remodeling.

Clinical samples from diseased and healthy groups are sources of exosomes, and abnormally increased and decreased biomolecules are screened based on databases or by high-throughput sequencing and proteomics analysis. Such studies are common and have identified valuable markers through the validation of clinical specimens. For example, low plasma levels of exosomal miR-150 (27) and miR-483 (250) and high levels of miR-596 (251) in patients with PAH suggested a poor prognosis; DQ593039 correlated significantly with diagnostic parameters in patients with CTEPH, and this piRNA showed potential value as a noninvasive screening agent (252). In practice, however, we do not know the donor cells, recipient cells, or specific mechanisms of abnormal exosomes in these disease groups, so further studies may be needed to elucidate the mechanisms of these markers in molecular, cellular, and animal models. This review has summarized the most recent and well-documented studies (Table 2 and Figure 4), which are fundamental to our understanding of exosomes, the development of vascular diseases, and the exploration of potential therapeutic approaches. Only by understanding the roles of these markers can we accurately use exosomes for disease diagnosis and monitoring.

**Table 2:** The role of exosomes in vascular disease.

Disease	Parent cells	Cargos	Recipient cells	Targets	Function	References
EH	ECs	miR-92a	VSMCs	Undefined	Leading to arterial stiffness	(75)
EH	Macrophages	miR-17-3p	ECs	ICAM1	Inhibiting endothelial inflammation	(77)
EH	ECs	miR-483-3p	SMCs	TGF-β, CTGF, ACE1	Protecting EC function	(78)
EH	AFs	ACE	VSMCs	Ang II	Promoting VSMC migration	(80)
EH	AFs	miR-155-5p	VSMCs	ACE	Attenuating VSMC proliferation and vascular remodeling	(81)
EH	AFs	miR-135a-5p	VSMCs	FNDC5	Promoting VSMC proliferation	(82)
EH	Monocytes	miR-27a	ECs	Mas	Impairing Ang-(1–7)-mediated vasodilation	(85)
EH, AS	LPS-treated macrophages	IL-6, IL-1β, TNF-α, MIP-2 and CAD, PAI-1, and Saa3 proteins	VSMCs	Nrf2/Keap	Increasing osteogenic markers and decreasing contractile marker expression	(88)
EH, AS	CUR-treated VSMCs	miR-92b-3p	VSMCs	KLF4, RUNX2	Attenuating vascular calcification	(94)
EH, AS	MT-treated VSMCs	miR-204, miR-211	VSMCs	BMP-2	Attenuating the calcification and senescence of VSMCs	(95)
AS	Endothelial colony-forming cells	miR-21-5p	ECs	SIPA1L2	Attenuating vascular endothelial injury, regulating lipid balance and activating autophagy	(108)
AS	Macrophages	miR-185-3p	ECs	Smad7	Suppressing cell proliferation and promoting cell apoptosis of vascular ECs	(113)
AS	DCs	miR-203-3p	Macrophages	Ctss/p38/MAPK	Inhibiting the atherosclerotic phenotype of BMDMs	(115)
AS	Macrophages	miR-146a	Macrophages	IGF2BP1, HuR	Decreasing macrophage migration and potential entrapment in the atherosclerotic plaque	(116)
AS	mDCs	miR-146a	HUVECs	IRAK	Protecting HUVECs from secondary stimulation; A negative feedback loop to regulate endothelial inflammation	(121)
AS	Ox-LDL-induced HUVECs	miR-155	Macrophages	Undefined	Increasing proinflammatory M1 macrophages and decreasing anti-inflammatory M2 macrophages	(122)
AS	ECs	miR-92a	Macrophages	KLF4	Inducing atheroprone phenotypes of macrophages	(123)
AS	CD137-modified ECs	IL-6	CD4^+^ T cells	NF-κB	Promoting the differentiation of Th17 cells; Accelerating inflammatory responses	(125)
AS	HUVECs with AdET-1 infection	miR-33	Macrophages	NR4A	Accelerating the effect of ET-1 on proinflammatory macrophage activation	(126)
AS	KLF5-induced VSMCs	miR-155	ECs	ZO-1 (zonula occludens-1)	Increasing endothelial permeability and enhancing atherosclerotic progression	(131)
AS	LSW-treated VSMCs	miR-145	HVUECs	PDCD4	Attenuating endothelial dysfunction	(132)
AS	Steatotic hepatocytes	miR-1	ECs	KLF4/NF-κB	Promoting endothelial inflammation and facilitating atherogenesis	(135)
AS	PLT	miR-25-3p	CVECs	Adam 10, NF-κB	Inhibiting ox-LDL-induced inflammation	(138)
AS	PVAT	miR-382-5p	Macrophages	BMP4-PPARγ-ABCA1/ABCG1, cholesterol transporter SR-A	Reducing the formation of macrophage foam cells	(139, 140)
AS	Nicotine-treated macrophages	miR-21-3p	VSMCs	PTEN	Increasing VSMCs migration and proliferation	(136)
AS	Macrophages	miR-186-5p	VSMCs	SHIP2, PI3K/Akt/mTOR	Promoting cell viability and invasion in VSMCs to accelerate AS	(146)
AS	Macrophages	miR-222	VSMCs	CDKN1B, CDKN1C	Promoting VSMC proliferation and migration	(148)
AS	PVAT	miR-221-3p	VSMCs	PGC-1α	Enhancing VSMC proliferation and migration	(150)
AS	Ox-LDL-treated macrophages	LIPCAR	VSMCs	CDK2, PCNA	Promoting the viability and migration of VSMCs	(151)
AS	Ox-LDL-treated macrophages	miR-106a-3p	VSMCs	CASP9	Promoting cell proliferation and repress cell apoptosis of VSMCs	(152)
AS	Ox-LDL-treated HUVECs	LINC01005	VSMCs	miR-128-3P/KLF4	Promoting VSMCs phenotype switch, proliferation, and migration	(153)
AS	High concentrations of glucose-treated macrophages	miR-486-5p	Macrophages	ABCA1	Accelerating spontaneous and diet-induced AS	(156)
AS	Insulin resistance adipocytes	shh	HUVECs	Ptch/Gli 1/TNF-α	Inducing vasa vasorum angiogenesis; Promoting plaque burden and plaque vulnerability	(158)
AS	Macrophages	miR-503-5p	HCAECs/HCASMCs	TGF-β/smad7/smurf1/smurf2	Inhibiting the proliferative and angiogenic functions of HCAECs; Promoting the proliferation and migration of HCASMCs	(161)
PAH	BOECs	TCTP	PASMCs	caspase-3	Inducing proliferation and reducing apoptosis	(186)
PAH	ASCs	miR-191	PAECs	BMPR2	Accelerating PAEC proliferation	(187)
PAH	MSCs	Undefined	PAECs/PASMCs	Wnt5a/BMRR2	Suppressing the vascular remodeling and right ventricular hypertrophy	(189)
PAH	PASMCs	miR-143	PAECs	Undefined	Inducing PAECs migration	(200)
PAH	PAECs	15-LO2	PAECs	STAT3	Affecting cell cycle distribution; Promoting PAEC proliferation and migration	(201)
PAH	PAECs	miR-181a-5p	PASMCs	KLF2, ETS-1, Notch4	Attenuating pulmonary hypertension	(209)
PAH	PAECs	miR-324-5p	PASMCs	KLF2, ETS-1, Notch4	Attenuating pulmonary hypertension	(209)
PAH	AFs	C3	Macrophages	C3ar1/C5ar1	Inducing inflammatory response	(195)
PAH	MSCs	Undefined	PASMCs	SIRT4, PDK4	Improving mitochondrial function	(198)

Stem cell therapy has become a promising strategy for the treatment of vascular diseases, yet stem cell transplantation is often complicated by graft-versus-host problems (55, 253, 254). Paracrine signaling *via* exosomes can take advantage of stem cell therapy while avoiding graft-induced complications (255), and the reversal of arterial muscularization and ventricular hypertrophy *via* exosomes has been reported (213). However, providing a safe and effective targeted therapeutic approach still requires further evidence of the feasibility and positive effects of exosomes in the treatment of vascular-related diseases.

## Author contributions

YR wrote the manuscript and produced the figures. HZ reviewed the manuscript. All authors contributed to the article and approved the submitted version.

## Funding

This work was supported by CAMS Initiative for Innovative Medicine (CAMS-I2M) [2016-I2M-3-006].

## Conflict of interest

The authors declare that the research was conducted in the absence of any commercial or financial relationships that could be construed as a potential conflict of interest.

## Publisher’s note

All claims expressed in this article are solely those of the authors and do not necessarily represent those of their affiliated organizations, or those of the publisher, the editors and the reviewers. Any product that may be evaluated in this article, or claim that may be made by its manufacturer, is not guaranteed or endorsed by the publisher.
